# MDT Clinics on a General Adult Acute Psychiatric Ward: Staff's Views and Person-Centred Care

**DOI:** 10.1192/bjo.2022.265

**Published:** 2022-06-20

**Authors:** Ademola Alalade, Zaha Kamran Siddiqui, Yiu Jong-Nam, Nikoletta Lekka

**Affiliations:** Sheffield Health and Social Care NHS Foundation Trust, Sheffield, United Kingdom

## Abstract

**Aims:**

Person-Centred Care (PCC) focuses on knowing the person behind the patient, engaging them as an active partner in their treatment, encouraging self-management and shared decisions. Inpatient multidisciplinary (MDT) clinics offer an opportunity for PCC by working collaboratively with service users (SU) in developing care plans. The aims of this project were to explore staff views and levels of satisfaction regarding the running of MDT clinics, to assess the quality and efficacy of changes made to MDT clinics, and to identify areas of practice which need improvement.

**Methods:**

In April 2021, MDT meetings of an acute inpatient clinical team were repurposed to 30-minute clinics with SU and relevant key professionals present, focusing on SU needs. Two staff surveys were completed in June and October 2021. Following the first survey, changes were made to the days clinics were run, attendance schedule, and staff allocation of responsibilities for efficient clinic running. In the second survey, a 14-question questionnaire was sent to all 48 staff members. The questions explored staff experience of MDT clinics. The measures were both qualitative and quantitative.

**Results:**

The overall response rate was 31.25%, of which 40% by medical and 40% by nursing staff. Staff reported there was a positive impact in the collaborative development of care plans, including improved SU involvement, increased involvement of families, improved contribution from different professionals, and formulations providing greater insight. They reported improved task orientation, directed responsibility for task completion within the team, and enhanced role and responsibility of the named nurse. They thought there was less time for 1:1 work, but that the “overall benefits are worth it”. Improved relationship with SU was reported by 85%, increased engagement with SU care by 93%, and identifying clear goals for care plans by 93%. Nevertheless, problems with planning and logistics were reported by 77%. Main challenges included time management especially with external visitors or combination of remote and face-to-face attendees, relatively poor attendance of CMHT and family members, difficulties with informing and preparing SU ahead of their clinic times, number of attendees, and dissemination of MDT care plans.

**Conclusion:**

Repurposing MDT meetings to MDT clinics focusing on SU needs has a positive impact in inpatient clinical practice. MDT clinic planning and improving the involvement of community teams and family members can contribute to an optimal purposeful inpatient admission. Conducting inpatient MDT clinics can be a crucial part of working collaboratively with SU and PCC.

